# Arterial Accesses in Coronary Angiography and Intervention—Review with a Focus on Prognostic Relevance

**DOI:** 10.31083/j.rcm2310331

**Published:** 2022-09-28

**Authors:** Christoph Langer, Rostislav Prog

**Affiliations:** ^1^Kardiologisch-Angiologische Praxis ● Herzzentrum Bremen, Senator-Weßling-Str 1a, 28277 Bremen, Germany; ^2^Klinik für Innere Medizin und Kardiologie, Hospital zum Heiligen Geist, Akademisches Lehrkrankenhaus der Universität Düsseldorf, Von-Broichhausen-Allee 1, 47906 Kempen, Germany

**Keywords:** transradial, snuff box, dorsal box, transulnar, transfemoral, coronary, angiography, PCI, classification, radial freedom

## Abstract

Arterial access in coronary angiography has always been an important issue. Convincing prognostic data from large randomized controlled trials (RCTs) in the first place but also safe performance of same-day-discharge after diagnostic and interventional procedures, improved patient comfort and cost-effectiveness led to a paradigm shift from the transfemoral approach (TFA) to the transradial approach (TRA) in several clinical situations. Consequently, today’s relevant guidelines recommend a radial-first strategy as default approach. However, there is still strong controversy among interventional cardiologists resulting in delayed spread of the TRA causing significant regional differences. One major critics point is the rate of postprocedural radial artery occlusion (RAO) after using the traditional puncture site at the proximal radial artery (pTRA) which was registered too high in certain centers. A new access using the distal radial artery (dTRA) in the area of the snuff box (SB) and the dorsal box (DB) has been proven to minimize RAO and enabling even complex interventions using 7F guiding catheters. Although, dTRA seems to be an advantageous option, this approach is still not widely used. This review—addressed to beginners and even advanced interventionalists—presents all arterial access routes in interventional cardiology. It focusses on those to be routinely preferred and also on the possibility to guide the puncture with ultrasound. Thereby, the various approaches, including the transulnar (TRU) but also the still relevant TFA approach, are discussed in detail. Thereby, we introduce our philosophy of “radial freedom” and a new classification for TRA.

## 1. Introduction

The first documented catheter access to the human heart in history is considered to be the self-experiment by Werner Forßmann in 1927—carried out from the cubital vein. Pioneering minimally invasive medicine in general, this was the basis for the further development of left cardiac catheterization, then requiring arterial access. With regard to the risk of prognostically relevant access site complications the location of the vascular puncture has, ever since, played an important role.

In fact, the technical development of selective coronary angiography introduced by F. Mason Sones [1] in the late 1950s was to be performed via the prepared distal brachial artery. However, in 1967 Melvin P. Judkins [2] reported on the advantages of the transfemoral approach (TFA) in coronary angiography which then was quickly adopted as the most popular vascular access for decades: Via the femoral artery, Andreas Grüntzig [3] performed the first balloon dilatation of a coronary stenosis in 1977 and Ulrich Sigwart the first implantation of a coronary stent in 1986. Not before 1989, a first feasibility study on transradial coronary angiography was published by Lucien Campeau [4] followed by Ferdinand Kiemeneij [5] in 1992 who reported on his first transradial coronary interventions.

Ever since, the transradial approach is increasingly propagated mainly because it leaves the patient with low periprocedural risk—especially after acute coronary syndrome and percutaneous intervention accompanied with intensified anticoagulation. Finally, this was supported by the RIVAL and RIFLE-STEACS studies. These randomized, parallel-group, multi-center trials prove transradial percutaneous coronary intervention (PCI) in high risk patients presenting with STEMI to be associated with a significantly lower mortality after 30 days and one year—owing from less relevant bleeding complications than if transfemorally performed [6, 7].

While this has already resulted in a higher proportion of transradial procedures among experienced interventionalists and junior cardiologists with concomitant lower bleeding rates, a risk-treatment paradox for access site selection remains: Femoral access is still too often used in patients with an increased risk of bleeding [8].

Today there is a huge body of comparing data on transradial vs. transfemoral access showing improved safety for the proximal radial artery and distal radial artery (p and dTRA) due to reduced major adverse cardiac events (MACE) in terms of significantly less vascular complications across the whole spectrum of patients with coronary artery disease (CAD) [9]. In 2017 the radial-first strategy as default access for coronary procedures was adopted by the ESC/EACTS Guidelines on Myocardial Revascularization as a class IA recommendation [10]. Currently, even the advantageous use of large radial sheaths in complex PCIs are supported [11].

**Fig. 1.**
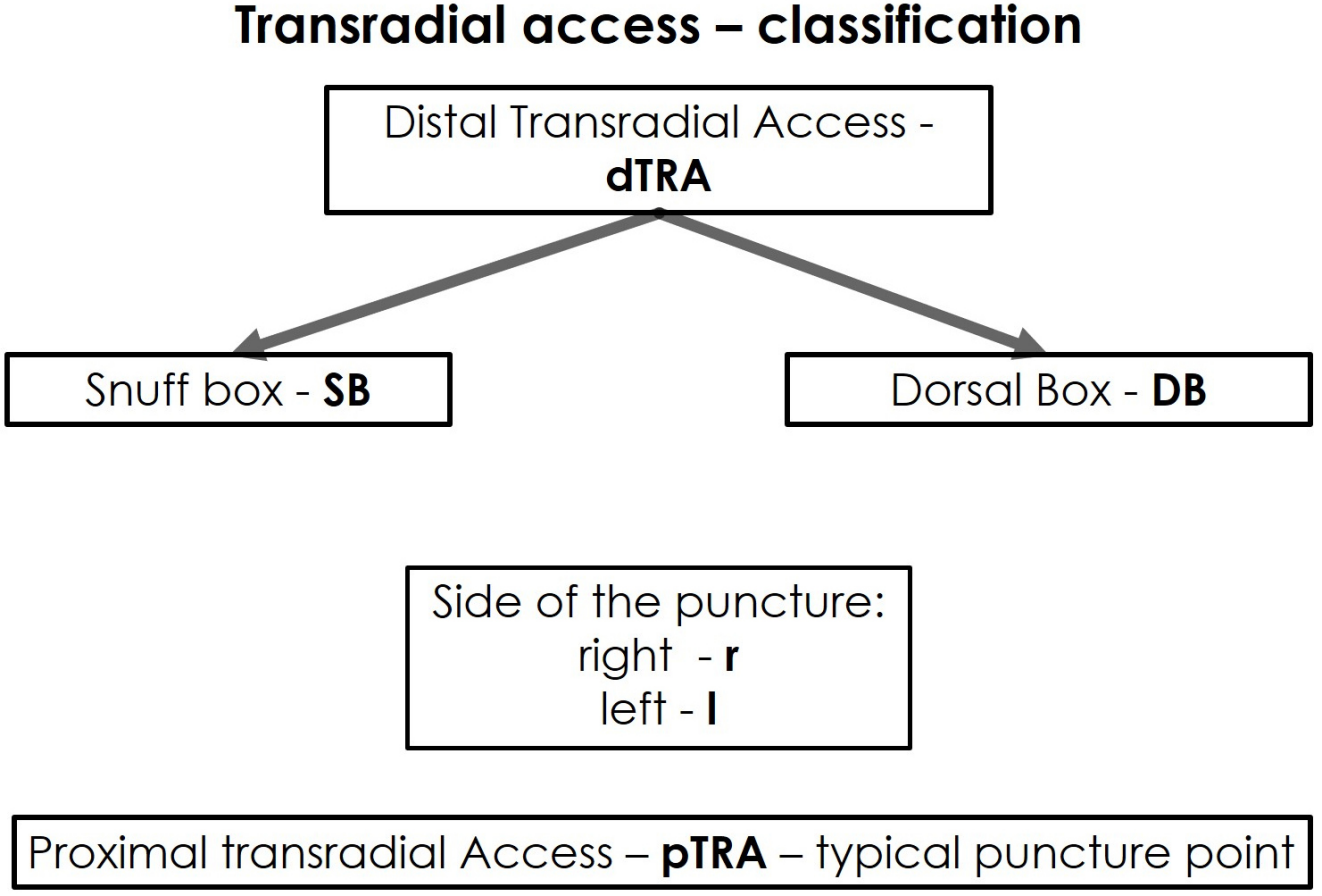
**Classification of the transradial access**.

However, when using the traditional radial puncture site—the proximal radial artery (pTRA)—radial artery occlusion (RAO) occurs in 5–8% (in meta analyses and up to >10% in certain centers) limiting future use of this safe route and, thus, feeding controversy among interventional cardiologists [12, 13, 14, 15]. Using a more distal radial segment—behind the offgoing superficial palmar arch—within the so called “snuffbox” (SB) or even further distal in the dorsal box (DB) has recently been proven to be less prone to occlude after puncture [16, 17, 18, 19] (Fig. 1). Despite these advancements, even in very experienced radial centers RAO rates remain high [20].

Another access site is the ulnar artery firstly introduced for left cardiac catheterization in 1949, and again, as an alternative to the transradial approach for coronary angiography in 2001 [21]. Yet, it has never widely been adopted although the AJULAR trial has proven transulnar interventions being non-inferior to the TRA and further reducing the need for cross over to TFA [22].

These and additional access sites for coronary angiography and intervention are closer described and discussed in the following. We focus on the ones to be preferred for prognostic reasons and present our philosophy of “radial freedom”.

## 2. Access Sites

### 2.1 Forearm Access Sites

The supplying vessels of the forearm are the often dominant radial and the usually thinner ulnar artery. However, there is a frequent variant arterial anatomy in the forearm—with regard to the radial artery (in consecutive patients scheduled for transradial heart cath) of up to 23%. Such could mean tortuous configurations unfavorable kinks, loops and bifurcations as well as a high bifurcating radial origin, hypoplasias, vascular spasms or a long stretch of unfavorably changed vascular system due to media sclerosis or atherosclerosis rarely leading to real stenoses. Farther distal in the hand the deep palmar arch is usually formed from the terminal part of the radial artery. The superficial palmar arch normally arises from the deep branch of the ulnar artery and connects it to the distal radial artery. Together both areas are the central part of the complex collateral rich arterial vasculature of the hand. While its precise function is not even completely understood yet [23] it prevents ischemia even in case of RAO and/or ipsilateral ulnar cannulation [24].

**Fig. 2.**
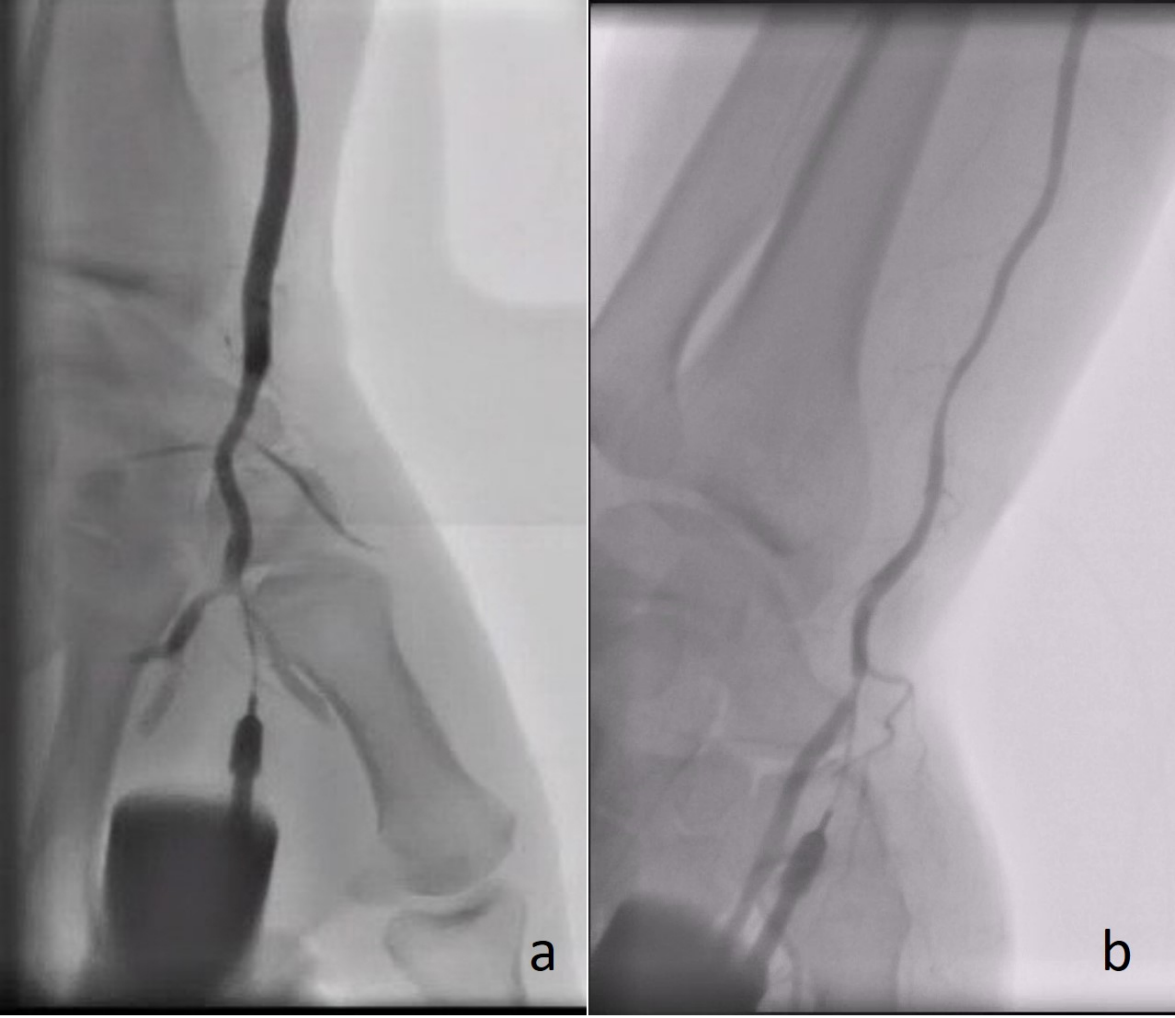
**Angiography demonstrating the radial artery using a plastic catheter directly after successful puncture of the (a) rDB and (b) rSB**.

Anyway which forearm access site is planned routine collateral testing (Allen’s and Barbeau test) is not supported by clinical evidence and is not recommended [25, 26, 27]. Crucial is adequate positioning of the arm and hand in order to tighten tissue and, thus, fix the artery. This facilitates rapid, precise and atraumatic puncture of the vessel. Given inability to cannulate the forearm arteries due to manifold reasons being the predominiant cause of failure for coronary procedures (57%) ultrasound-guidance is an option to improve first attempt success rate and decrease failure rate significantly [28]. Existent ultrasound expertise is a necessity for this. The puncture technique used also here is the “modified Seldinger technique” performed with a hollow needle. This way a straight 0.025” floppy guide wire is advanced into the vessel over which a “sheath” can be placed into the access artery. A sheath is a hollow plastic cannula, usually between 4 and 7 French (1 French = 0.33 mm), which provides a well-sealed working access to the arterial lumen throughout the procedure. In case of resistance during wire manipulation in the vessel the sheath should not be inserted but checked under fluoroscopy before inserting the sheath. Then, the intraluminal position maybe proven, by the pulsatile arterial backflow, blood pressure curve control or angiographically using the plastic part of an intravenous tube (Fig. 2). If the sheath is placed successfully administration of spasmolytic cocktails and heparin is possible according to the local “radial protocol”.

In the event that a 0.035“ wire cannot be advanced to the proximal arterial segments despite a correctly positioned sheath, an unfavorable vascular anatomy as enumerated above must be assumed and correspondingly visualized angiographically. Current investigations on the transradial approach registered such difficulties in 10–14% of cases then potentially leading to complications and a varying failure rate [29, 30].

#### 2.1.1 Proximal (“Traditional”) Transradial Access—pTRA

**Fig. 3.**
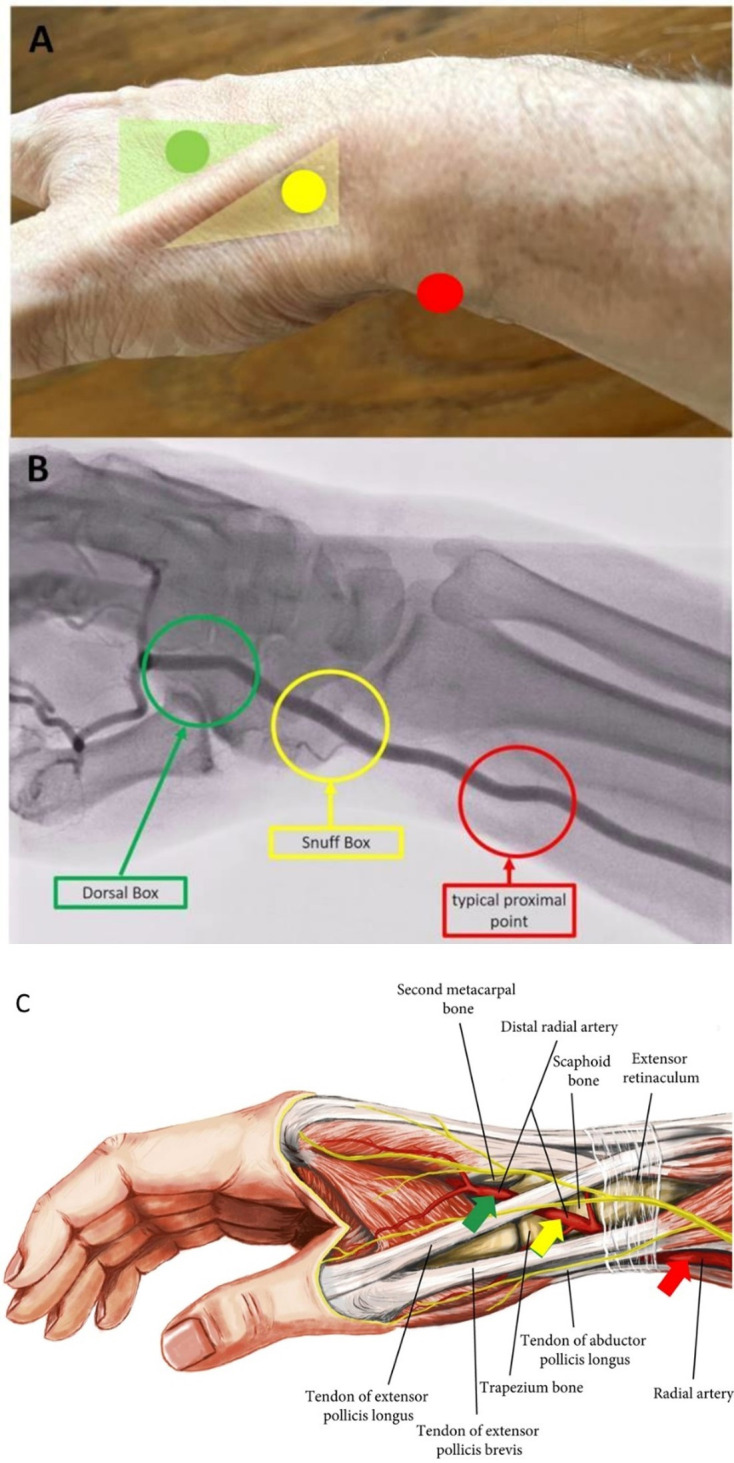
**Transradial puncture sites of the radial artery**. In (A) Topography, (B) Angiography and (C) Anatomy (adapted to [31]). In (B) the arcus palmaris superficialis and profundus is not recognizable.

The pTRA is the traditional radial puncture site and in the area of the distal radial head, about 2 cm proximal of the palpable processus styloideus radii which should be palpated (Fig. 3, Ref. [31]). For access via the pTRA the arm is externally rotated and positioned parallel to the trunk. Following hyperextension of the wrist (dorsiflexion of the hand) the artery with its desired puncture area is located relatively close under the skin.

#### 2.1.2 Distal Transradial Access—dTRA

The youngest development in arterial approach is the distal transradial access (dTRA) performed from the SB and DB (Fig. 3). Basic studies investigating the diameter of the distal radial artery via ultrasound confirmed, that the arterial diameter in the SB and DB is significantly smaller comparing to the pTRA (2.33 ± 0.43 mm and 2.04 ± 0.38 mm, respectively, vs. 2.76 ± 0.48 mm; all *p *< 0.05) [32]. Kim *et al*. [33] examined the diameter of the left distal radial artery as a significantly larger vessel in men (2.65 ± 0.46 mm) compared to women (2.40 ± 0.53 mm). Moreover, an anatomical cadaver study showed a medium to very high variation in the lumen diameter of the distal radial artery in the SB and that the average lumen diameter in male cadavers was greater than that in female (2.79 ± 0.35 mm vs. 2.26 ± 0.66 mm respectively).

Cardiological use of the dTRA was first described by Babunashvili in 1995 [34]. Finally, A. L. Kaledin and F. Kiemeneij introduced the dTRA as a default access site for coronary angiography and intervention [35, 36]. Having published this technique as video on YouTube© and Twitter© facilitated a uniquely rapid spreading of the dTRA in interventional cardiology and radiology worldwide (https://www.youtube.com/watch?v=-If5oAF0KJo). Thus, the first based on social media initiated medical trial was started by three investigators who met via Twitter© [16]. Besides promising results for dTRA-feasibility the TRIANGLE and other registries showed low RAO rates for dTRA which were confirmed by randomized trials comparing dTRA with conventional pTRA as statistically significant [17, 18, 37, 38, 39].

However, the dTRA includes two different access sites. For a better differentiation and comprehensive understanding of the dTRA—including its two anatomical and topographic subdivisions, thus, requiring different puncture techniques and postoperative management—we hereby propose a new classification for dTRA using a new terminology (Fig. 1).

2.1.2.1 dTRA—“Snuff Box” (SB)

The term “snuff box” (SB; in French “tabatière” or for the anatomically correct term: “fovea radialis”) was first mentioned in 1850 by the anatomist Marie Bichat [27]. It describes the small triangular area of the medial hand. The SB is the area the radial artery segment distal of the pTRA site runs through after receiving the arcus palmaris superficialis at the dorsal side of the hand. It ends in the arcus palmaris profundus after giving off a thumb branch. This topographic small triangle—deepening on the dorso-lateral surface of the hand and wrist presents with the following borders—proximal: radial styloid process, distal: base of 1st metacarpal bone, floor: scaphoid and trapezium, medial: tendon of the extensor pollicis brevis muscle and lateral: tendon of the extensor pollicis longus muscle [40] (Fig. 3).

2.1.2.2 dTRA—“Dorsal Box” (DB)

After leaving the SB the distal radial artery runs postero-laterally between the metacarpal bone I and II across the dorsal surface of the hand. Thus, the puncture is possible in the area we call the “Dorsal Box” (DB) located distal to the SB and the tendon of the extensor pollicis longus muscle, respectively. While SB is assigned to the front part of the dTRA, DB is assigned to its rear part. With the thumb spread, the DB can be recognized as an acute-angled triangle. The borders of them are medial: the tendon of the extensor pollicis longus muscle, lateral: the metacarpal bone II and floor: the first dorsal interosseus muscle (Fig. 3).

2.1.2.3 dTRA—Patient Selection and Preparation

**Fig. 4.**
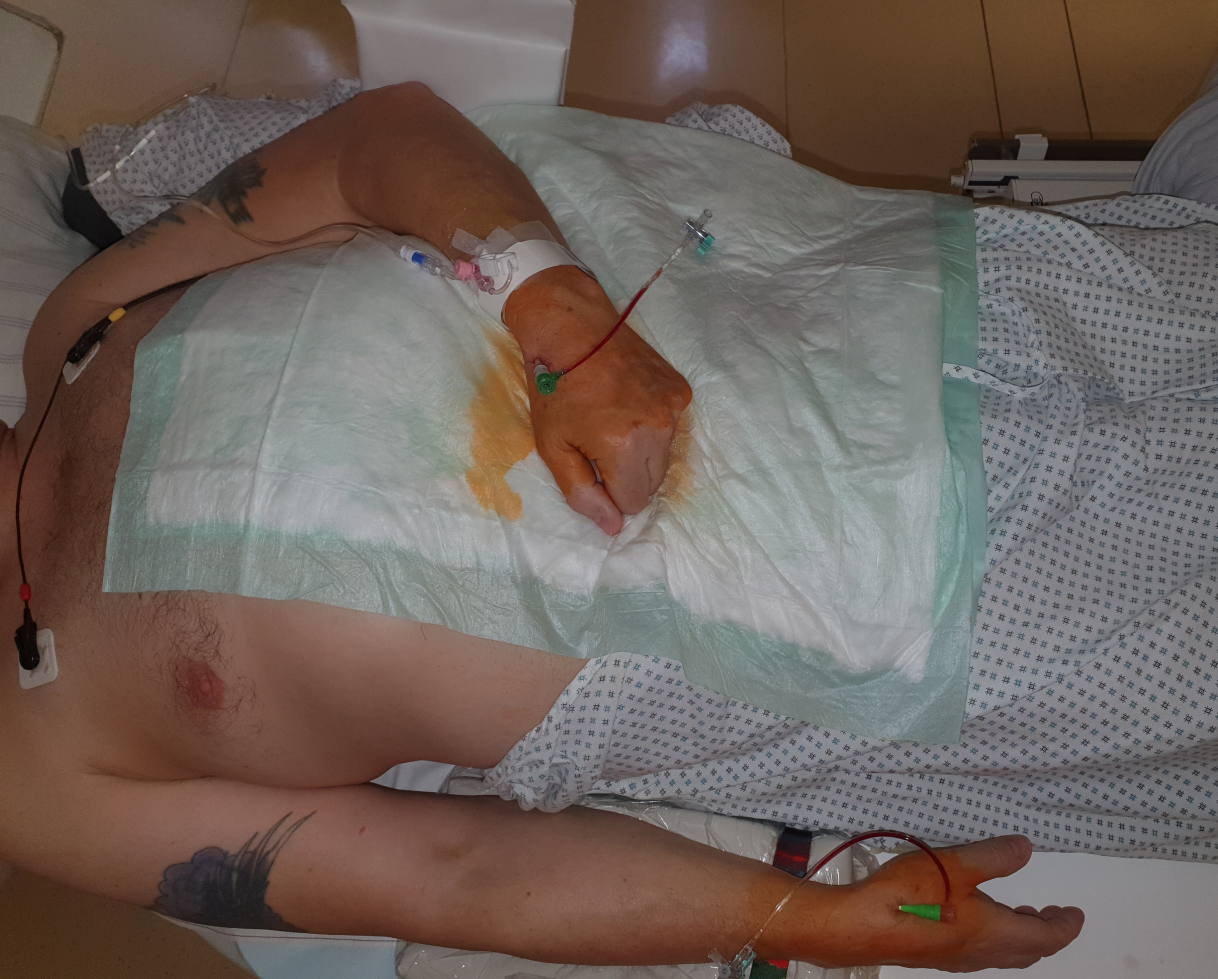
**Patient’s positioning for a bilateral dTRA (rDB and left snuff box (lSB)) chronic total occlusion-percutaneous coronary intervention (CTO-PCI)**. Almost physiologically both hands come to lie slightly overstreched next to or on the body.

Suitable for the dTRA are patients with a palpable pulse in the area of the SB and/or slightly more distally in the DB. The patient’s preparation does not differ from the pTRA. The same boards or table extensions can be used to position the patient’s arm. For the right-sided access the patient’s arm is positioned physiologically along the body. The hand should be aligned with the dorsal surface in the SB/DB area ventrally to the operator. A relaxed slightly overstretched position of the hand with the thumb parallel to the other fingers helps to shift the distal radial artery more superficially and to facilitate the puncture. For the left-sided access the arm is positioned using special boards and/or large cushions so that the forearm is positioned on the lower abdomen or left groin area (Fig. 4). The left-sided dTRA allows for the operator to stand on the right side of the patient and thereby have a significantly better ergonomic body position during the procedure.

Overall, the distal approach does not require major changes in the workflow of a cardiac cath lab which proficiently practices the transradial approach via pTRA. It is recommended to use a sterile “4-hole” drape, which—in the rare case of a cross-over—allows a quick change of puncture site. The left femoral hole of the sterile drape may then avoid significant shifting of the drape to the right.

2.1.2.4 dTRA—Puncture in the “Snuff Box” and “Dorsal Box”

The same puncture sets and techniques, which are used for pTRA, are applied for dTRA. After the puncture sites have been disinfected, local anesthesia with 3–5 mL of the usual cathlab anesthetic is administered subcutaneously. In order to choose the optimal puncture site an ultrasound-guided puncture may be useful during the learning curve. The puncture angle when using SB is approximately 45° to the skin and approximately 25–30° from lateral to medial. Since the vessel (as in pTRA) runs flat in case of DB the puncture angle should be about 30–45°. In our own study we showed significantly shorter access time at the SB puncture point compared to the more distal DB (81 ± 74 sec vs. 99 ± 84 sec, respectively; *p* = 0.01), without any differences in the puncture success and cross-over between the groups [16].

2.1.2.5 dTRA—Catheter Selection

Catheters used for dTRA are basically the same as for pTRA. However, due to the smaller vessel diameter of the arm flow path and its occasional vasospasm, many centers practice the so-called one-catheter concept—the use of one catheter that both coronary ostia can usually be intubated with. The mechanical stimulus but also dye volume and radiation exposure were proven to be reduced by using only one instead of two coronary catheters [41]. From our point of view suitable for diagnostic purposes is the Tiger II while, e.g., the Amplatz Left (AL)-guiding catheter maybe cautiously used for both coronary osita in case of multi-vessel PCI [42].

For patients taller than 185 cm, the usual catheter length of 100 cm may not be sufficient since the distal puncture causes a loss of around 5–9 cm in the catheter length. For such patients it is recommended to use diagnostic and guide catheters of the most common shapes with a length of 110, 118 or 125 cm. In our experience, patients taller than 200 cm are usually successfully examined via the dTRA.

2.1.2.6 dTRA—Limitations

Limiting for the dTRA is a non-palpable pulse in the SB and/or DB area. As with the pTRA, however, in certain clinical situations a puncture of the dTRA must be attempted and successfully carried out despite a weak or even no pulse. Here, ultrasound-guidance maybe an option. After using pTRA another limitation may be a proximally located RAO. This, in fact, may be recanalized via the dTRA which should then enable a coronary procedure [34]. We do not recommend performing a primary PCI in a myocardial infarction via dTRA during the “learning curve”.

2.1.2.7 dTRA—Hemostasis Concept

With the SB approach, hemostasis can be achieved quite easily. However, insufficiently high pressure on the vessel above its bony floor can result in complete obstruction of the radial artery segment within the SB. There is no bony floor in DB, but due to their muscle and fat mass and smaller artery diameter, effective hemostasis is still easy to achieve.

**Fig. 5.**
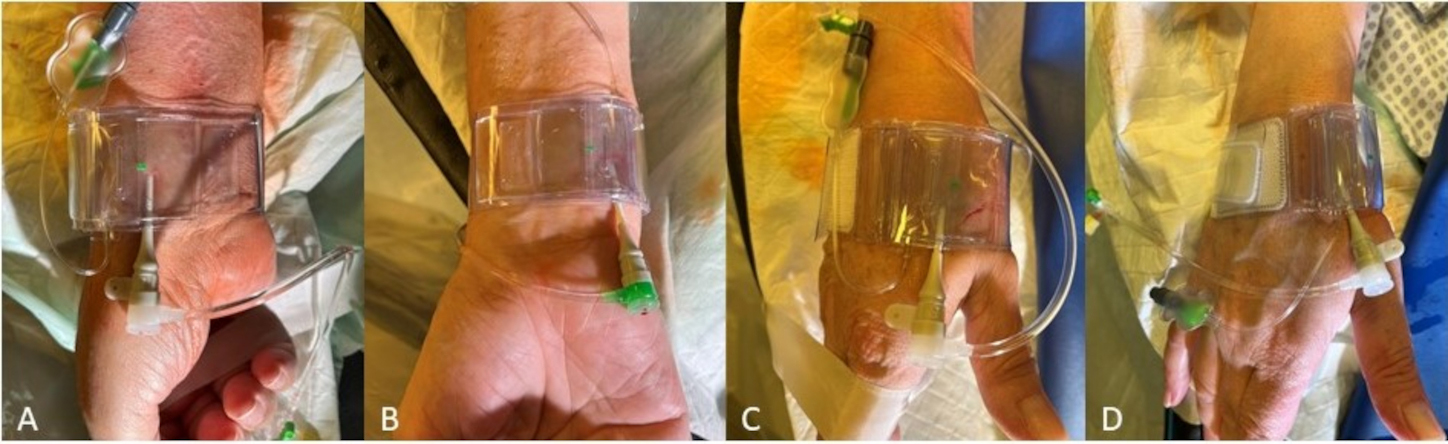
**Hemostasis after rDB access using a modified TR-Band® (Terumo Corporation, Tokyo, Japan) for (A) pTRA, (B) TRU (supplying air hose pointing upwards after rotated 180°), (C) dTRA DB and (D) dTRA SB**.

The closure devices established for pTRA usually fit for the hemostasis after dTRA too (e.g., the modified TR-Band®; Terumo Corporation, Tokyo, Japan) (Fig. 5). Thereby, after DB access the TR-Band providing an air cushion may best be used as modified TR-Band® by removing of the hard-plastic plate. The dTRA dedicated device, Prelude Sync distal® (Merit Medical Systems, South Jordan, UT, USA), shows a very good anatomical fitting and successful hemostasis after SB access but may appear limited in placement, covering and stability in our experience case of DB access.

According to current understanding, the concept of patent hemostasis is primarily responsible for low RAO rates and is therefore of great importance. Patent hemostasis is an individual, non-occlusive compression that prevents bleeding from the puncture site (hemostasis) but allows blood flow through a radial artery that is still open. This should be possible (after pulling the sheath) by using air cushion compression (TR band) with only 2 mL more air than needed to just stop the bleeding.

The shortest possible compression time is just as important as open hemostasis. In general, lower rates of RAO in dTRA than in pTRA are potentially explained by reduced time-to-hemostasis and maintained flow at the wrist during hemostasis [43]. Here, collateral testing (e.g., plethysmography) may help to optimize patent hemostasis and/or identify vascular occlusion—under or after compression.

We were able to remove a hemostasis device after DB access in selected patients (no oral anticoagulation (OAC), only diagnostics with 2000 IE heparin) after 30–40 minutes and without any complications. However, we recommend using the usual pTRA hemostasis protocols for everyday routine to simplify the postprocedural workflow and to pay the meticulous attention to patent hemostasis. As with pTRA, there is no increased bleeding complication rate with dTRA [16, 19, 35].

#### 2.1.3 Proximal (“Traditional”) Ulnar Access

As known from current ultrasound-based investigations the ulnar artery—dependent on race, gender, presence of certain cardiovascular risk factors and anatomical variability—is often the straighter forearm artery presenting with fewer morphological variants. It can even be of larger diameter than the radial artery [21, 44, 45, 46, 47]. Furthermore, there are fewer alpha-receptors in the ulnar than in the radial artery, which is why the ulnar artery is supposed to be less prone to vascular spasm [48, 49].

When planning the transulnar access, the puncture site is located about 3 cm proximal to the flexor crease along the axis with the clearest pulsation of the ulnar artery. With an externally rotated arm and hyperextension of the wrist (dorsiflexion of the hand similar to the pTRA-access) the ulnar puncture site is still seated comparatively deeper than the radial artery. Both, ulnar artery and nerve run close to each other, the nerve medially which is therefore susceptible to irritation. Postprocedural hemostasis can also be achieved with the TR band rotated 180° after placement in the area of the ulnar puncture site (Fig. 5).

### 2.2 Traditional Access Sites

#### 2.2.1 Brachial Access Site according to Mason Sones

Especially in long term diabetes mellitus or advanced renal failure both forearm arteries may be more or less atherosclerotic or present with media sclerosis potentially making them fragile and hard to pass. The brachial artery is of a larger caliber and often of a straight course. A high take off going brachial bifurcation is even less frequent than kinks and loops of the lower or aberrant side vessels of the upper third of the brachial artery.

Even before the femoral access was established the distal brachial artery served as a promising access site for coronary angiography. Unlike other approaches for coronary angiography, Mason Sones suggested preparing this puncture site by cutting through the overlying tissue down to the vessel [1]. This technique was left, has never been reintroduced as default access but still serves as reserve approach in certain cases. The fact that the brachial artery supplies the forearm arteries can make complications at the puncture site of the (distal) brachial artery crucial.

#### 2.2.2 Femoral Access Site (Common Femoral Artery)

The target vessel for TFA is the common femoral artery. It arises from the external iliac artery on both sides, which in turn arises from the common iliac artery distal to the aortoiliac bifurcation. The aorta running above can be divided into the abdominal and thoracic aorta. Both the iliac tract and the aorta can be straight and free of any pathological wall changes to the other extreme of extensive kinks (and iliac loops) leading to unfavorably angled visceral artery branches but also aneurysms, heavy wall calcifications and/or thrombotic adhesions. Peripheral artery disease in terms of relevant atherosclerotic stenoses and total occlusions finds its manifestations in 40% within the iliacal arteries whereas only in 10% within the upper limb arteries. In such cases the transfemoral approach may be very difficult to overcome or even impossible without prior peripheral intervention. Iliac kinks and loops may require stiff wires or/and long sheaths that allow passage of the iliac artery and improved maneuverability of the catheters toward the coronary ostia. Given the poor prognosis of vascular complications in the groin identifying the inguinal ligament is critical for puncture below the common femoral artery. Puncturing above the retroperitoneal external iliac artery should be avoided. Besides palpation and finding the line between the Spina iliaca anterior superior and Tuberculum pubicum, alternatively, the optimal puncture site can be localized by fluoroscopy: medial to the equatorial plane of the femoral head. Moreover, ultrasound was adopted as part of the routine in different cath labs.

### 2.3 Other Access Sites

#### 2.3.1 The Distal Ulnar Access

The approach via the superficial palmar branch of the ulnar artery initially described by Roghani-Dehkordi [50], is not a default but may serve as an alternative access. The puncture site is on the medial aspect of the palmar surface 1 cm distal and lateral to pisiform bone and about 2.5 cm distal to the wrist crease. According to the author the procedure is successful in only about one-third of all patients. Prognostic criteria for the successful access were older age, obese, athletes, workers, RAO and weak radial pulse. Technical aspects are sedation, sublingual nitroglycerin and spasmolytic cocktail, because palmar artery is highly spastic, unfractionated heparin (5000 IU) as anticoagulant and local anaesthesia. The patient’s arm is positioned at 45 degrees to the trunk. The hand is held in a mildly extended position (dorsiflexion of about 20–30 degrees) without hyperextension to prevent the arteria from stretching in order to collapse. Otherwise, postprocedural haemostasis could be achieved due to wrist hyperextension (up to 90 degrees) for 15 minutes followed by local compression and—if 6F sheath was used—with a TR-band. Described complications included hand ecchymosis, self-limited hematoma of proximal forearm or distal arm, transient paresis and hyposthesia with complete recovery within 1–2 weeks. Major complications (including ulnar occlusion, thrombosis, infection, hand dysfunction) were not observed [50].

#### 2.3.2 The Superficial Temporal Artery (STA) Access 

The access route via the superficial temporal artery (STA) is neither a default access but an example for an alternative approach as described in a case report by Ruzsa [51]. The patient had bilateral subclavian artery occlusions and an internal carotid artery occlusion on the left side. Initial attempts through left and right femoral arteries had failed. After the left temporal scalp was shaved, sterile prepped and draped left STA was punctured under ultrasound guidance and the same technique as the radial artery approach requires. After a 5F 7.5 cm radial sheath was advanced into the common carotid artery unfractionated heparin (5000 IU) and nitroglycerine (250 mg) were administered. Postprocedural haemostasis was easily achieved by a haemostatic patch followed by a local compression and gaze covering [51].

## 3. Discussion

Based on a growing body of data comparing TRA with TFA as well as significant international differences in the choice of the vascular access especially in ACS all current European and US-American guidelines recommend the transradial approach as default access route. This is to primarily reduce mortality due to vascular complications leading to relevant bleedings, in both, emergency and elective settings [8, 10, 27, 52, 53, 54, 55]. Moreover, we know about its generally advantageous use in today’s increasingly propagated outpatient area, its cost-effectiveness in general and last not least patient satisfaction resulting from examination comfort, faster mobilization and untouched pubic settings [56, 57, 58].

Despite the convincing prognostic value of the transradial access it is its feasibility which often remains controversial even among academic cardiologists. After the initial transbrachial vascular access had been abandoned at an early stage TFA was undisputed for a long time. Frequent arguments for the access from the groin are still the larger vessel cross-section of the common femoral artery, which lets the puncture technique here appear easier to perform. Besides, certain interventions requiring ≥7F catheters would require this access route, anyway. Accompanying arguments against TRA are manifold. Frequent concerns are smaller diameters of the access arteries, the allegedly higher anatomical variability of the upper limb flow path, an allegedly lower support when using conventional guiding catheter bends and that patients after TRA temporarily develop a dysfunction of the hand.

At least one technique—either TFA or TRA—is well established in daily cath lab routine worldwide. In fact, they are feasible and usually (in cases with normal anatomy) easy to perform. However, the approach via the upper limb flow path includes the subclavian artery and brachiocephalic trunk. Especially, the latter presents with a certain anatomic variability possibly showing a buckling course, which—if additionally calcified—may be difficult to overcome. Moreover, both vessel segments are closely related to the vertebral and carotid arteries, which can branch off at an unfavorable angle. Furthermore, the anatomy of the aortic arch includes the three types defining the angle of the supra-aortic branches, how they hit the aorta and how catheters may reach the aortic bulb.

Many laboratories perform coronary angiographies and PCIs from the left radial artery as first choice, assuming that the catheter deliverability and stability is similar to femoral and superior to right transradial access with a theoretically reduced incidence of stroke. The latter could not be proven in a meta analysis including 12 studies which compared the left- with the right-transradial approach. In fact, the left-transradial access was associated with slightly less dye and lower fluorescence time [59].

On the other hand, TFA via tortuous and calcified iliacal arteries or aortic pathologies may similarly cause considerable friction then leading to limited maneuverability of the catheter possibly demanding extra devices. Whichever access route is chosen—TRA or TFA—the aortic arch, the shape and dimension of the ascending aorta and its bulb may vary due to elongation, dilatation or even dissection and, thus, influences catheter maneuverability as well. Additionally, even a normal let alone an abnormal coronary outlet configuration may challenge the interventionalist beyond what is normal.

Since all procedures are associated with a certain learning curve, the TRA can pose a challenge for the “femoralist”. This is mainly due to its initially unfamiliar anatomy, anatomical variants (that also occur here), and more frequent vascular spasms still allowing a success rate of around 95% [60]. Despite the smaller arterial diameters of the forearm latest data focusing on complex interventions, such as PCIs of the left main and CTO requiring a 7F guiding catheter still indicate very good results of the TRA [10, 61, 62, 63]. Furthermore, the comparison of TFA with TRA procedures showed coming from the forearm is at least not inferior with regard to the incidence of periprocedural stroke [64, 65, 66]. Irrespective of this, the more comfortable patient positioning during transradial procedures especially in case of obesity or orthopaedic problems helps to guide patients. Right-handed individuals examined from the left TRA may still use the dominant limb. However, latest data proves TRA not to be associated with postprocedural hand function impairment [67]. Due to the quick hemostasis after TRA in general (quicker after dTRA) patients are discharged even earlier from hospital [68]. The comparatively early mobilization after transradial procedures often warrants less discomfort. Hence, patients do not remember their heart cath as an unpleasant event.

The biggest criticism and well-known complication of the pTRA, albeit mostly asymptomatic, is RAO with rates reported >10% in certain centers and before the effective prophylaxis could be defined as today [14, 15]. Underlying mechanisms are associated with intimal injury followed by stasis due to inadequate compression leading to thrombus and fibrotic obliteration. In general smallest sheath/catheter possible supported by the Slender technique and periprocedural anticoagulation with unfractionated heparin of at least 5000 IE or 50 U/kg BW are recommended [27, 69, 70, 71]. With the establishment of prophylactic measures RAO-rates could be reduced to <3% [12, 72, 73, 74].

Today RAO should definitely be reduced to a minimum by guaranteeing the above described “patent hemostasis” [75]. Concomittant ipsilateral ulnar artery compression was proven to support radial artery patency [72]. In our facilities we start reducing the air compression by 1 mL after 1 hour, then again by 1 mL after 30 minutes and again by 1 mL after another 30 minutes. It is often possible to remove the TR-band after about two hours.

Suggested alternatives to the pTRA reach from getting back to the femoral artery or to the ulnar artery. Even more advanced is the use of the dTRA performed within the SB or DB—where the radial artery is of a thinner caliber. Using the radial artery here means distal of the off going superficial arcus palmaris which is assumingly responsible for a low RAO-rate [16, 35, 40]. However, if pTRA and its postprocedural compression is performed properly as by high volume radialists involved in the DISCO trial RAOs can be reduced to a minimum of <1% [19].

However, after transradial procedures a patent radial artery may present with intimal hyperplasia and, thus, should be used cautiously for surgical myocardial revascularization. With regard to other future procedures that may be required by the patient (such as the operation of a dialysis shunt) cardiologists should consider the appropriateness of using the right radial artery for access [23]. For such purposes the left radial artery maybe used in right-handed people and vice versa.

### “Radial Freedom”

**Fig. 6.**
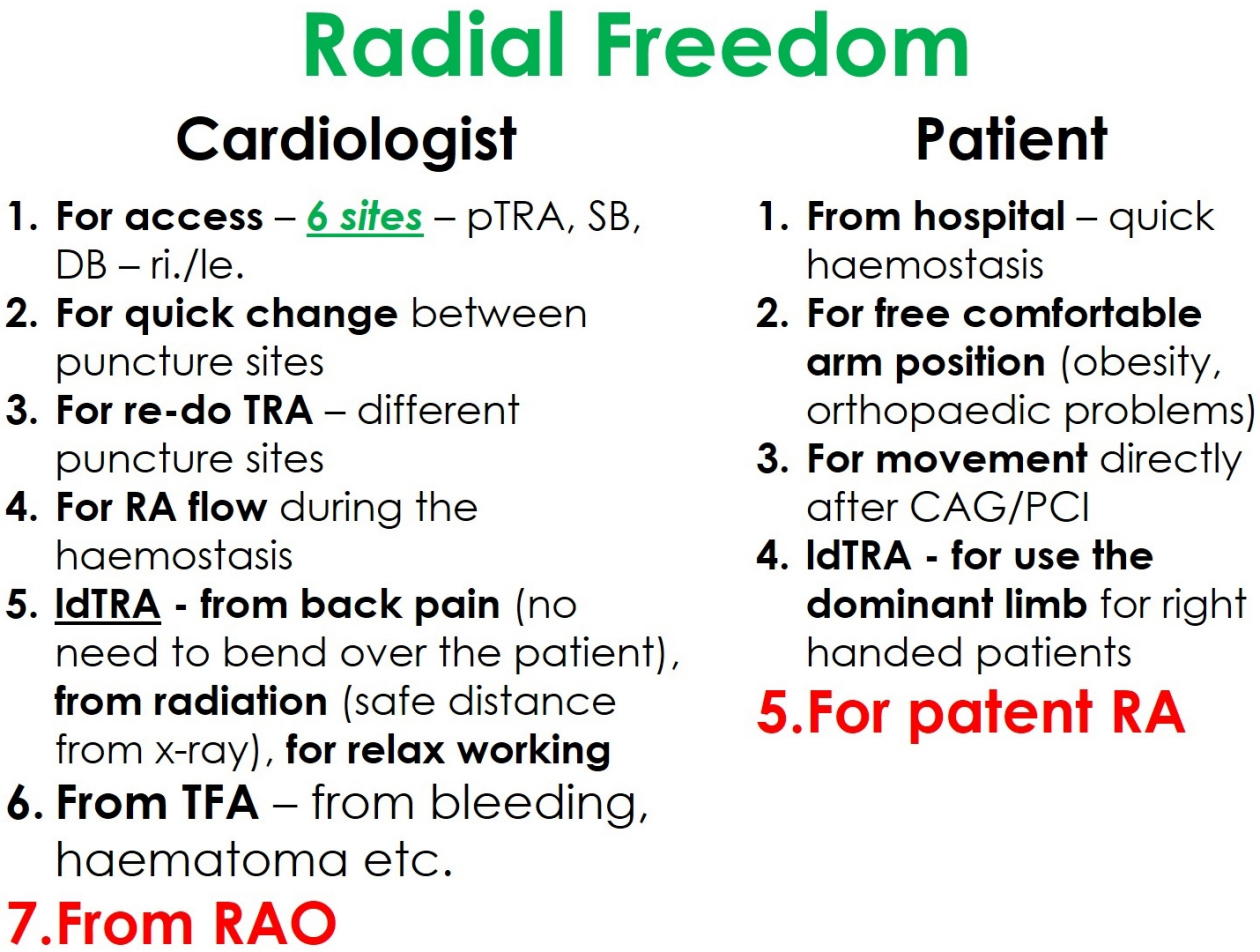
**The philosophical concept of “Radial Freedom”**.

The new distal radial access sites give an additional motivation for the adoption of the transradial access for coronary procedures. Thus, we practice our philosophy called “Radial Freedom”, which combines the advantages of different radial puncture sites (Fig. 6).

“Radial freedom” means the new dTRA—SB and DB—not to compete with the pTRA, but being part of an overall concept. Take “freedom” as it is—having the choice of the best or most appropriate access. For interventionalists, in fact, there are six radial puncture sites—plus two ulnar. Despite potential disadvantages most studies found TRU to be noninferior compared to TRA [76]. Other studies support the ipsilateral ulnar approach as viable after a failed transradial approach or in the case of RAO. However, this data is insufficient to recommend the ipsilateral ulnar over the contralateral transradial approach [77, 78]. In the rare event of failure, a quick switch to a different puncture site is feasible. This way, freedom from complications can be achieved at least minimizing prognostically unfavorable complications typically associated with TFA.

In case of a short-term necessary repeated TRA, “Radial Freedom” also offers the option of choosing a different transradial access route so that the first radial puncture site can recover [79]. Moreover, “Radial Freedom” goes so far that transradial access with a CTO-PCI on both sides is also possible. Finally, patent hemostasis guaranteed by pTRA or dTRA can bring us freedom from RAO, which could even be recanalized by dTRA if needed [34].

After years of practicing “Radial Freedom”, and occasionally also considering the proximal ulnar artery, we are convinced that primarily the patients, but also the interventional cardiologist, benefit from this concept.

However, representing TRA means taking responsibility. We point out that transradial catheterization must be practiced proficiently in daily routine and before being carried out safely in emergencies. We also want to mention the controversy about the Campeau paradox [80, 81]—that at least certain groups which modified their routine and switched from TFA to TRA as default access then registered significantly more complications from TFA. Irrespective of this, recently published registry data on relevant bleeding and 30-day mortality in consecutive STEMI patients show that a vascular occlusion system has no significant advantage after TFA [82]. Against this background, it must be pointed out that the ability to use TFA should be retained and therefore trained and practiced further. In the daily routine of a cath lab predominantly practicing TRA, TFA means to have a reserve arterial access. Ultrasound guidance for TFA, especially in case of large-bore accesses, should always be an option.

## 4. Conclusions

In view of the comparable limiting anatomical variability of the transradial and transfemoral approaches, current prognostic data, but also patient satisfaction and cost-effectiveness speak strongly for the further introduction of transradial approaches as the first choice for coronary interventions. Thereby, interventionalists should individually decide which access route from the arm to use. However, confident skills of the transfemoral access should further be guaranteed by all interventional cardiologists. Considering certain cases where a transfemoral approach is required, comprehensive training to reduce complications should be standard in all cath labs.
